# Effect of 100% Orange Juice and a Volume-Matched Sugar-Sweetened Drink on Subjective Appetite, Food Intake, and Glycemic Response in Adults

**DOI:** 10.3390/nu16020242

**Published:** 2024-01-12

**Authors:** Stephanie Robayo, Michaela Kucab, Sarah E. Walker, Katherine Suitor, Katherine D’Aversa, Olivia Morello, Nick Bellissimo

**Affiliations:** School of Nutrition, Toronto Metropolitan University, 350 Victoria Street, Toronto, ON M5B 2K3, Canada

**Keywords:** 100% orange juice, sugar-sweetened beverages, sugars, satiety, food intake, appetite, glycemic response, blood glucose, normal-weight adults

## Abstract

Dietary recommendations to reduce the consumption of free sugars often group 100% fruit juice with other sugar-containing beverages. The objective of this study was to determine the effect of consuming 100% orange juice compared to an orange drink on next-meal food intake (FI), glycemic response, average appetite, emotions, and sensory characteristics in normal-weight adults. Thirty-six normal-weight adults (age: 26.8 ± 0.9 years) consumed, in random order and at least 5 days apart, three 240 mL test beverages as follows: (a) 100% orange juice, (b) orange drink, or (c) water. Subjective sweetness and pleasantness were determined immediately after test beverage consumption. Glycemic response, average appetite, and subjective emotions were measured every 15 min for 60 min. Food intake was determined at a pizza lunch 60 min later. Rest-of-day glycemic response and energy intake (EI) were determined using a continuous glucose monitor and food record, respectively. Lunch FI (*p* = 0.054) and total EI (*p* = 0.01) were both lower after 100% orange juice compared with the orange drink. Caloric compensation was 84% after 100% orange juice and −25% after the orange drink (*p* = 0.047). Average appetite was not significantly different between the test beverages (*p* > 0.05). Blood glucose iAUC adjusted for available carbohydrate was lower after 100% orange juice compared with the orange drink (*p* < 0.001). Rest-of-day blood glucose concentrations were lower after 100% orange juice compared with the orange drink (*p* = 0.03) and water control (*p* < 0.001). In conclusion, consumption of 100% orange juice as a preload resulted in higher caloric compensation, lower total daily EI, and lower blood glucose concentrations compared to the orange drink.

## 1. Introduction

Decreasing the intake of free sugars to 10% or less of daily energy intake (EI) is suggested by the World Health Organization to reduce the risk of unhealthy body weight and dental caries [1]. Consistent with these recommendations, some national dietary guidelines recommend reducing the intake of sugars from all sources [2]. Yet, because these recommendations do not distinguish the source of free sugars and are based primarily on data from observational studies, the physiological mechanisms underlying any observed effects cannot be readily determined.

There is a significant amount of literature showing that sugars suppress food intake (FI) in adults, but the effects are dependent on the source, dose, and meal timing. For example, in young men, 50 g of glucose [3], or a sucrose-sweetened yogurt [4] suppressed FI 60 min later. In contrast, high amylose/amylopectin cornstarch-sweetened drinks (75 g) failed to reduce FI after 60 min [5], suggesting that carbohydrates eliciting a higher glycemic response are associated with greater suppression of subjective appetite and lower FI 1–2 h later in short-term studies [5,6]. However, sucrose-sweetened beverages provided immediately before or with lunch appear to add to EI [7]. This effect extends beyond sugar-sweetened beverages (SSBs), where within meal consumption of regular cola, orange juice, and 1% milk increased meal EI compared to diet cola or water [8].

100% fruit juices are readily available nutrient-dense options containing naturally present sugars and, apart from fiber, can provide micronutrients and bioactive compounds at doses similar to those found in whole fruits [9,10,11]. Unlike 100% fruit juices, SSBs are beverages with caloric sweeteners added during processing [12]. In Canada and the United States, orange juice ranks among the most frequently consumed fruit juices [13]. In addition, 100% orange juice is a source of micronutrients and can be supplemented by the manufacturer with several key shortfall nutrients such as calcium and vitamin D [14]. However, there has been a decrease in consumption of 100% orange juice in Canada and the United States [13,15,16]. Given the ongoing concern that excess energy consumption derived from free sugars may contribute disproportionately to the obesity epidemic [9,17], there is a need for further investigation of different sources of sugars such as 100% orange juice and SSBs on FI regulation.

The associations among free sugars, body weight, and metabolic risk may be mediated by the source of free sugars. A recent meta-analysis of controlled feeding trials highlighted the importance of source and energy control of fructose-containing sugars on adiposity [18]. In addition trials, consumption of 100% fruit juice, derived 100% from fruits with no added sugars at 10% or less of EI, was associated with a moderate decrease in body weight and body mass index (BMI), while consumption at >10% of EI had no effect on body weight or BMI [18]. Conversely, fruit drinks derived from fruit juices or fruit flavoring with added sugars and mixed fructose-containing sources were associated with moderate elevations in body weight and BMI in addition trials [18]. Furthermore, a synthesis of prospective cohort studies found that sweetened fruit juices were associated with an increased risk of type 2 diabetes, but not 100% fruit juice [19]. Furthermore, meta-analyses of prospective observational studies and randomized controlled trials did not find a relationship between 100% fruit juice and increased risk of cardiovascular disease [20,21].

Limited experimental studies have directly compared the effects of 100% orange juice to an SSB on satiety and FI or explored potential mechanisms of action. Evidence from experimental trials is needed to understand the physiological effects of consuming 100% orange juice compared to commonly consumed beverages containing added free sugars or water [22], contributing the level of granularity needed to inform dietary guidance. Therefore, the objective was to determine the effect of consuming 100% orange juice compared to an orange-flavored SSB on next-meal FI, subjective appetite, glycemic response, subjective emotions, and sensory characteristics in normal weight adults. We hypothesized that consuming 100% orange juice as a preload would increase satiety, mood, and suppress FI through its effect on post-prandial glycemia.

## 2. Materials and Methods

### 2.1. Participants, Inclusion and Exclusion Criteria

Adults aged 18 to 45 years of age were recruited via online classified advertisements, social media, and by word-of-mouth. To meet the inclusion criteria, adults were required to have BMI between 18.5 and 24.9 kg/m^2^, to have been habitual breakfast consumers [23], and to have been willing to consume the study treatment foods and beverages. Adults were excluded if they were on a diet or had food allergies, sensitives, or aversion to any of the food or beverages in this study. Furthermore, adults were excluded if they had significant weight fluctuations within the past 6 months or a previous diagnosis of diabetes, pre-diabetes, gastrointestinal disease, liver disease, kidney disease, or a metabolic disorder. Participants who received a score of ≥11 points on an eating habits questionnaire were considered as restrained eaters and excluded [24].

### 2.2. Study Consent

A telephone screening interview provided initial eligibility criteria. Participants who passed the telephone screening were scheduled to attend an in-person information and final screening session at the laboratory to review study protocols and obtain written informed consent. Anthropometric measures were obtained, and participants were familiarized with test day instruments and procedures during the information and final screening session. Height was measured to the nearest 0.5 cm using a wall-mounted stadiometer (SECA, 803, 220 height rod, Hamburg, Germany). Body weight was recorded to the nearest 0.1 kg (Bod Pod, Life Measurement Instruments, Concord, CA, USA). Fat mass and fat-free mass were determined using the Bod Pod, employing adult-specific body fat equations tailored for sex and ethnicity (Bod Pod, Life Measurement Instruments, Concord, CA, USA). To accommodate dietary preferences, eligible participants were asked to rank their top two flavor preferences from a selection of three pizza options including: three-cheese, pepperoni, or deluxe (Selection Brand, Mini Pizza, Metro, ON, Canada). This study protocol received approval from the Toronto Metropolitan University Research Ethics Board (REB #2021-177) and was registered at clinicaltrials.gov (NCT05012046).

### 2.3. Experimental Design

Following a within-subjects, repeated measures study design, eligible participants completed three test sessions at least 5 days apart. On three separate mornings, participants arrived at the laboratory at 8:00 am following a 10 to 12 h fast, except for a small amount of water which was permitted up to 1 h before arriving. On the morning of each test session, participants completed a baseline questionnaire to verify adherence to the fasting protocol and other pre-test requirements.

Following completion of the baseline questionnaire, a Dexcom G6 (Dexcom Inc.©, San Diego, CA, USA) continuous glucose monitoring system (CGM) was inserted onto the abdomen of the participants. Participants were then provided a standardized breakfast of 2% milk (237 mL, 120 kcal, Neilson, Saputo Inc., Montreal, QC, Canada), a strawberry breakfast bar (37 g, 130 kcal, Selection), and cereal (27 g, 110 kcal, Cheerios, General Mills Inc., Minneapolis, MN, USA). Participants were required to consume the entire breakfast. After participants completed the standardized breakfast, a two-hour waiting period was commenced to allow for the Dexcom G6 to warm up as previously reported [25,26].

After the two-hour period and the collection of baseline measures, participants consumed, one of the three isovolumetric (240 mL) treatments within five minutes, including (1) 100% orange juice (105.6 kcal, 25.9 g of carbohydrate, Tropicana Pure Premium Original, No Pulp, Chicago, IL, USA), (2) orange drink (119.1 kcal, 29.8 g of carbohydrate, Orange Soda, Compliments, Sobeys Inc., Stellarton, NS, Canada), or (3) a water control (0 kcal, 0 g of carbohydrate, Nestle Pure Life^®^, North York, ON, Canada). Participants consumed each test beverage only once during the study. Test beverages were served cold in opaque containers with lids and straws. Subjective appetite, subjective emotions, and subjective physical well-being were determined at baseline (0-min) and at 15 min intervals for 60 min. Glycemic response was measured by finger-prick at baseline (0-min), and 15, 30, 45, and 60 min post-treatment consumption. Sensory characteristics, including pleasantness and sweetness of the test beverages, were collected post-consumption (5 min). At 60 min, participants were served an ad libitum pizza lunch assess lunch FI. After completion of the laboratory test session, participants were sent home with the Dexcom G6 CGM inserted to measure rest-of-day glycemic control and a rest-of-day food and a physical activity record to complete and return at their next test session.

### 2.4. Experimental Procedures

#### 2.4.1. Food and Water Intake

An ad libitum pizza lunch was used to assess lunch FI at 60 min post-treatment, as we previously reported [27,28,29,30]. Participants were individually seated in a private cubicle in our sensory room adjacent to the kitchen. Participants were provided with ad libitum water for 30 min and a fresh tray of pizza every 10 min for 30 min. Each tray consisted of three mini-pizzas, two of the participant’s first choice, and one of their second choice. Each pizza was weighed (g) prior to serving. Participants were reminded to eat until they were comfortably full. Any leftover pizza was weighed and recorded. The frozen three-cheese pizza delivered 260 kcal (106 g, Selections, Toronto, ON, Canada), while the pepperoni offered 250 kcal (106 g, Selections, Toronto, ON, Canada), and the deluxe option supplied 240 kcal (106 g, Selections, Toronto, ON, Canada), as per the nutrition facts table.

The amount of pizza consumed (g) was determined by subtracting the total weight of leftover pizza from the total weight of served pizza. FI at lunch (kcal) was then computed using the weight of consumed pizza (g) and the information available on the nutrition facts panel. The pizzas were used in this study due to the absence of an outer crust and consistent macronutrient and energy density [31]. Water consumption (g) was determined by the change in weight measured before serving and upon completing the ad libitum lunch. Cumulative FI (kcal) was determined by the sum of energy consumed from the standardized breakfast, test beverage, and the ad libitum pizza lunch. Caloric compensation (%) was calculated by comparing the ad libitum caloric intake after the control preload (water) versus the ad libitum caloric intake after each high energy preload (100% orange juice or orange drink): compensation (%) = (control treatment lunch intake (kcal) − treatment preload intake (kcal)/treatment preload kcal × 100) [32,33].

#### 2.4.2. Rest-of-Day EI and Physical Activity

Upon completing each study session, participants completed a paper-based food and activity record and were instructed to document all foods and beverages consumed during the remainder of the day along with any physical activity. To estimate rest-of-day EI (kcal) and macronutrient intake (total carbohydrate, total fat, and protein), each food record was analyzed using the Food Processor SQL software (Version 9.8, 2005, ESHA Research, Salem, OR, USA). Total daily EI (kcal) was computed from the sum of EI from the standardized breakfast, the test beverage, the ad libitum pizza lunch, and the rest-of-day food record. Physical activity energy expenditure was assessed by determining the metabolic equivalent of task (METs) based on the participant’s reported non-sedentary activities. The METs value was multiplied by the participant’s weight (in kilograms) and the reported duration of activity (in hours) [34].

#### 2.4.3. Post-Prandial Glycemic Response

Capillary blood glucose samples were collected from participants’ fingertips via a single-use, auto-disabling lancet device (Safe-T-Pro Plus, Accu-Chek, Roche Diabetes Care^®^, Laval, QB, Canada). Three large blood drops were collected into a sodium fluoride/potassium oxalate blood collection tube for measurement (Terumo America Inc., Tustin, CA, USA). Blood samples were immediately analyzed for glucose concentration in the YSI 2950D Glucose Analyzer (YSI Inc., Yellow Springs, OH, USA). Changes in glucose concentrations in the blood typically precede changes in interstitial fluid, especially during times when glucose is changing rapidly (i.e., post-beverage consumption) [35]. Therefore, capillary blood glucose via the finger-prick method was used during the acute phase (over 60 min post-treatment consumption), and the Dexcom G6 CGM was used to observe glycemic response for the remainder of the day.

#### 2.4.4. Rest-of-Day Glycemic Response

Rest-of-day glucose concentrations were collected using the Dexcom G6 CGM system, which collects glucose measurements from the interstitial fluid. Dexcom CGM is a widely used and validated instrument for tracking glycemic response trends [25,26]. Dexcom CGMs were inserted by the participants on their abdomen following the manufacturer instructions and with the assistance of a trained research assistant. The Dexcom G6 provided glycemic response data every 5 min for 11 h. The data collection period for the rest-of-day glycemic response phase started at 1:00 p.m. (i.e., after test sessions were completed) and continued until 11:59 p.m. of each test session day. A total of eleven time-points representing the mean from each hour of blood glucose data for the participant were analyzed for rest-of-day glycemic response.

#### 2.4.5. Subjective Appetite, Subjective Emotions and Sensory Characteristics

Satiety encompasses the sensation of fullness experienced from the cumulative inhibitory signals triggered by the ingestion of food and beverages [36]. Paper-based 100 mm line visual analogue scale (VAS) questionnaires were used to determine the effect of the test beverages on subjective appetite, emotions, physical well-being, and sensory characteristics. Participants marked an ‘X’ along the 100 mm line anchored by two opposing statements based on how they currently felt, as previously reported [30].

A motivation-to-eat VAS questionnaire was used to determine subjective appetite [27,30,37,38]. Each question of the motivation-to-eat VAS questionnaire addressed one of the four dimensions as follows: desire to eat, hunger, fullness, and prospective food consumption. An average appetite score was then calculated as previously reported [27,30,37,38].

Subjective emotions were evaluated using a VAS questionnaire consisting of thirteen questions, as previously reported [28,29]. An average subjective emotion score (mm) was calculated from the thirteen VAS questions as previously reported [30,31]. An additional VAS question was administered to assess physical well-being by inquiring ‘how well do you feel right now?’ anchoring the response from ‘not well at all’ to ‘very well’. Sensory characteristics of the test beverages were evaluated via pleasantness and sweetness using a VAS question at 5 min post-treatment consumption. The test beverage pleasantness question asked, ‘how pleasant did you find the beverage you consumed?’ with anchors ‘not pleasant at all’ to ‘very pleasant’. The test beverage sweetness question asked, ‘how sweet did you find the beverage you consumed?’ with anchors ‘not sweet at all’ to ‘very sweet’.

### 2.5. Statistical Analyses

Statistical analyses were performed using SAS version 9.4 (SAS Institute Inc., Cary, NC, USA). A repeated-measures two-factor analysis of variance (ANOVA), using the PROC MIXED procedure, was used to determine the effect of treatment (i.e., test beverage), time, and treatment-by-time interaction on change from baseline subjective average appetite, subjective emotions, subjective physical well-being score, and blood glucose concentrations. To determine the effect of treatment on blood glucose incremental area under the curve (iAUC), FI measurements (lunch FI, cumulative FI, rest-of-day EI, rest-of-day macronutrient intake (grams), and total EI), water intake, subjective appetite iAUC, subjective emotions iAUC, rest-of-day physical activity (METs), and sensory characteristics, a one-factor repeated measures ANOVA was used. To control for multiple comparisons when main effects were significant, a Tukey–Kramer’s post hoc analysis was employed. The effect of treatment on caloric compensation was analyzed using a paired *t*-test. Blood glucose, subjective average appetite, and subjective emotion iAUC were calculated using the trapezoid method [39]. Pearson correlation coefficients were used to examine the associations among subjective average appetite iAUC, subjective average emotions iAUC, lunch FI, and blood glucose iAUC over 60 min. All data are reported as mean ± standard error of the mean (SEM). A *p*-value < 0.05 was considered statistically significant.

## 3. Results

### 3.1. Participant Characteristics

Thirty-six adults (18 males and 18 females, age: 26.8 ± 0.9 years) completed the study (Table 1). All participants consumed all treatment beverages. Thirty-four participants (17 males, 17 females) completed post-prandial blood glucose measurements for 60 min. Thirty-one participants (15 males, 16 females) had complete rest-of-day blood glucose measurements.

### 3.2. Food and Water Intake

There was a main effect of treatment on lunch FI (*p* = 0.054), cumulative FI (*p* = 0.007) and total EI (*p* = 0.01) (Table 2). Lunch FI was lower after 100% orange juice (*p* = 0.053) compared with the orange drink, but there was no difference when compared with water. Cumulative FI was lower after 100% orange juice (*p* = 0.02) and water (*p* = 0.01) compared with orange drink. Total EI was lower after 100% orange juice (*p* = 0.008) compared with orange drink, but there was no difference when compared with water. There was a main effect of treatment on caloric compensation (*p* = 0.047). Mean caloric compensation after the 100% orange juice preload was 84.0% ± 38.0 and after the orange drink preload was -24.7% ± 40.1. Ad libitum water intake was not affected by treatment (*p* = 0.17) (Table 2).

### 3.3. Rest-of-Day EI

There was a main effect of treatment on rest-of-day EI (*p* = 0.04). However, after Tukey–Kramer adjustment, no significant difference was observed for treatment on rest-of-day EI (Table 2). There was a main effect of treatment on rest-of-day intake of carbohydrates (*p* < 0.05), but not dietary fat or protein (*p* > 0.05). Rest-of-day intake of carbohydrates (in grams) was lower after 100% orange juice (*p* = 0.02) compared with orange drink.

### 3.4. Rest-of-Day Physical Activity

There was no effect of treatment on rest-of-day physical activity METs (*p* = 0.70). 

### 3.5. Post-Prandial Blood Glucose Concentrations

**Figure 1:**
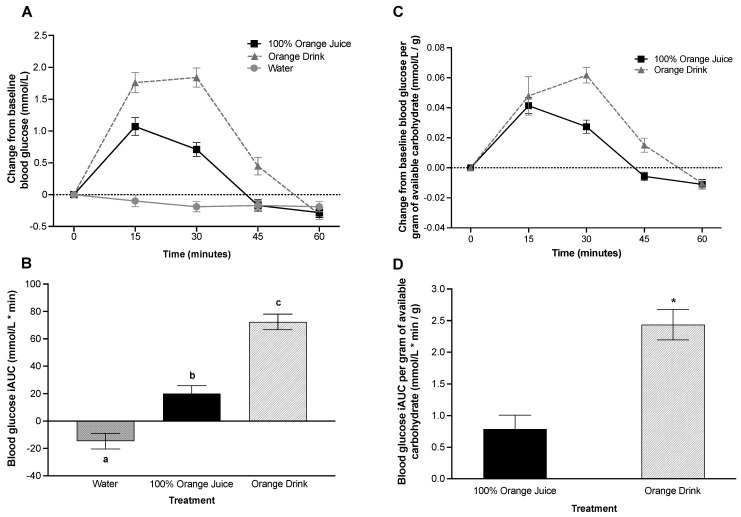
(**A**) Effect of treatment on change from baseline blood glucose (mmol/L) over 60 min. Blood glucose was affected by treatment (*p* < 0.001), time (*p* < 0.001), and treatment-by-time interaction (*p* < 0.001). Blood glucose concentrations were lower after 100% orange juice compared with orange drink at 15 min (*p* = 0.001), 30-min (*p* < 0.001), and 45 min (*p* = 0.005), but not at 60-min (*p* > 0.05). (**B**) Effect of treatment on blood glucose incremental area under the curve (iAUC) (mmol/L * min) over 60 min. Blood glucose iAUC was affected by treatment (*p* < 0.001). Blood glucose iAUC was lower after 100% orange juice (*p* < 0.001) and water (*p* < 0.001) compared with orange drink. Different letters represent significant differences between treatments (*p* < 0.05). (**C**) Effect of treatment on change from baseline blood glucose per gram of available carbohydrate (mmol/L/g) from test beverages over 60 min. Blood glucose per gram of available carbohydrate was affected by treatment (*p* = 0.004), time (*p* < 0.001) and treatment-by-time interaction (*p* = 0.015). Blood glucose concentrations per gram of available carbohydrate were lower after 100% orange juice compared with orange drink at 30 min (*p* = 0.003), but not at 15, 45, or 60 min (*p* > 0.05). (**D**) Effect of treatment on blood glucose iAUC per gram of available carbohydrate (mmol/L * min/g) from test beverages over 60 min. Blood glucose iAUC per gram of available carbohydrate was affected by treatment (*p* < 0.001). Blood glucose iAUC was lower after 100% orange juice (*p* < 0.001) compared with orange drink. Asterisks represent significant differences between treatments (*p* < 0.05). All values are means ± SEM, *n* = 34.

Blood glucose was affected by treatment (*p* < 0.001), time (*p* < 0.001) and treatment-by-time interaction (*p* < 0.001) over 60 min post-treatment consumption. Blood glucose was lower after consumption of 100% orange juice (*p* < 0.001) and water (*p* < 0.001) compared to orange drink, and lower after water (*p* < 0.001) compared with 100% orange juice. Blood glucose concentrations were lower after 100% orange juice compared with orange drink at 15 min (*p* = 0.001), 30 min (*p* < 0.001), and 45 min (*p* = 0.005), but not at 60 min (*p* > 0.05) (Figure 1A). There was a main effect of treatment on blood glucose iAUC (*p* < 0.001). Blood glucose iAUC was lower after 100% orange juice (*p* < 0.001) and water (*p* < 0.001) compared with orange drink (Figure 1B). Blood glucose iAUC was lower after water compared with 100% orange juice (*p* < 0.001).

### 3.6. Post-Prandial Blood Glucose Concentration per Gram of Available Carbohydrate in the Test Beverage

Blood glucose per gram of available carbohydrate in the test beverage over 60 min was affected by treatment (*p* = 0.004), time (*p* < 0.001), and treatment-by-time interaction (*p* = 0.015). Blood glucose was lower after 100% orange juice compared to the orange drink (*p* = 0.004). Blood glucose concentrations per gram of available carbohydrate were lower after 100% orange juice compared with orange drink at 30 min (*p* = 0.003), but not at 15, 45, or 60 min (*p* > 0.05) (Figure 1C). There was a main effect of treatment on blood glucose iAUC per gram of available carbohydrate over 60 min (*p* < 0.001). Blood glucose iAUC per gram of available carbohydrate was lower after 100% orange juice compared with orange drink (*p* < 0.001) (Figure 1D).

### 3.7. Rest-of-Day Glycemic Response

**Figure 2:**
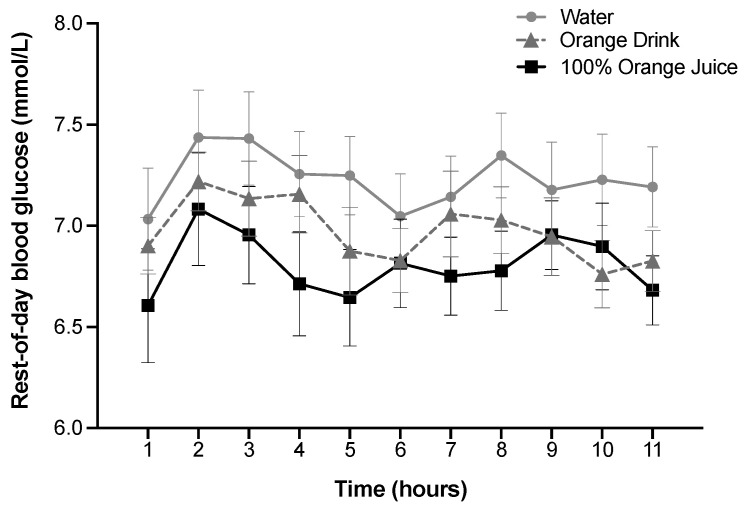
Effect of treatment on rest-of-day blood glucose (mmol/L) over 11 h. Blood glucose was affected by treatment (*p* < 0.001), but not time or treatment-by-time interaction (*p* > 0.05). Rest-of-day blood glucose was lower after 100% orange juice compared with the orange drink (*p* = 0.03) and water (*p* < 0.001), and lower after orange drink compared with water (*p* = 0.002). All values are means ± SEM, *n* = 31.

Rest-of-day blood glucose over 11 h was affected by treatment (*p* < 0.001), but not time or treatment-by-time interaction (*p* > 0.05). Rest-of-day blood glucose was lower after 100% orange juice compared with the orange drink (*p* = 0.03) and water (*p* < 0.001), and lower after orange drink compared with water (*p* = 0.002) (Figure 2).

### 3.8. Subjective Appetite and Subjective Emotions

**Figure 3:**
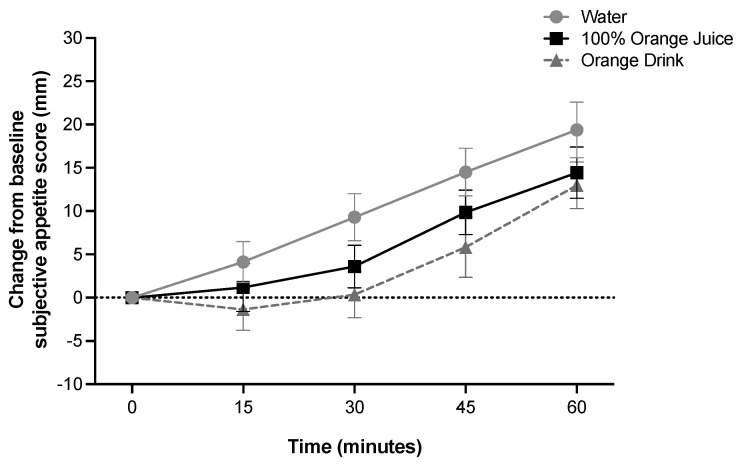
Effect of treatment on change from baseline subjective appetite scores (mm) over 60 min (two-factor ANOVA with Tukey–Kramer’s post-hoc test to account for multiple comparisons). Subjective appetite scores were affected by time (*p* < 0.001), but not treatment or treatment-by-time interaction (*p* > 0.05). All values are means ± SEM, *n* = 36.

Subjective average appetite was affected by time (*p* < 0.001), but not treatment, and there was no treatment-by-time interaction (*p* > 0.05) (Figure 3). Subjective average appetite was lower at 15 min compared to 30 min, 45 min, and 60 min (*p* < 0.001), lower at 30 min compared to 45 and 60 min (*p* < 0.001), and lower at 45 min compared to 60 min (*p* < 0.001). Subjective average appetite iAUC was not affected by treatment (*p* > 0.05). Subjective average emotions were not affected by treatment, time, or treatment-by-time interaction (*p* > 0.05). Subjective emotions score iAUC was not affected by treatment (*p* > 0.05). Subjective wellness score was not affected by treatment, time, or treatment-by-time interaction (*p* > 0.05).

### 3.9. Sensory Characteristics of Treatments

**Figure 4:**
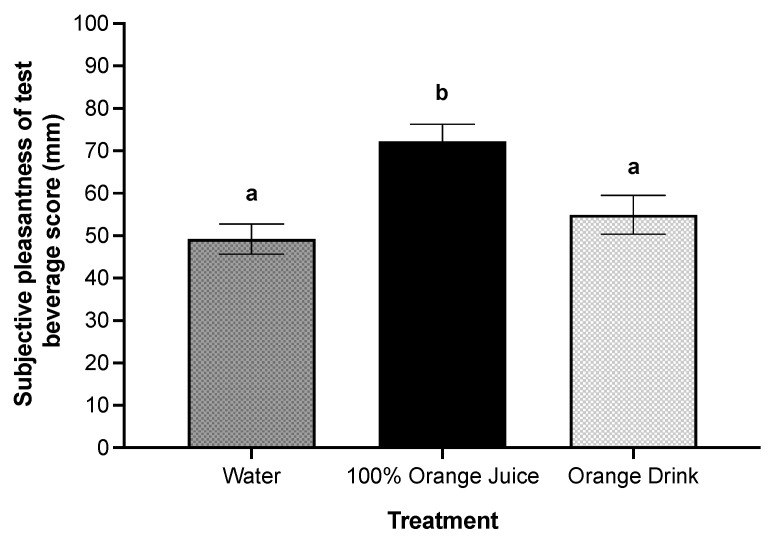
Effect of treatment on subjective pleasantness scores (mm) post-treatment consumption. There was a main effect of treatment on subjective pleasantness of the test beverage (*p* < 0.001). Subjective pleasantness was higher after 100% orange juice compared with orange drink (*p* = 0.009) and water (*p* < 0.001). All values are means ± SEM, *n* = 36. Different letters represent significant differences between treatments (*p* < 0.05).

There was a main effect of treatment on subjective sweetness (*p* < 0.001). Subjective sweetness was higher after orange drink compared to 100% orange juice (*p* = 0.003) and water (*p* < 0.001), and higher after 100% orange juice compared to water (*p* < 0.001). There was a main effect of treatment on subjective pleasantness of the test beverages (*p* < 0.001). Subjective pleasantness was higher after 100% orange juice compared with orange drink (*p* = 0.009) and water (*p* < 0.001) (Figure 4).

### 3.10. Correlations

Blood glucose iAUC was positively associated with subjective average appetite iAUC after 100% orange juice (r = 0.39, *p* = 0.02, *n* = 35) but not after orange drink or water. Subjective average emotions iAUC was inversely associated with average appetite iAUC after orange drink (r = −0.51, *p* = 0.002, *n* = 36) but not after water or 100% orange juice. Subjective sweetness was positively associated with lunch FI only after orange drink (r = 0.34, *p* = 0.04, *n* = 36) but not after 100% orange juice or water.

## 4. Discussion

The present study addressed a crucial knowledge gap in our understanding of how the source of free sugars affects subjective appetite, FI, and glycemic response in young adults. Our results show that 100% orange juice suppressed lunch FI, cumulative FI, and total day EI compared with the orange drink. While rest-of-day EI was lower after 100% orange juice, this finding was not statistically significant when compared to the orange drink. Although the glycemic response over 60 min was higher after both caloric treatments compared with the water control, glycemic response was lower after 100% orange juice compared with the orange drink.

Our findings emphasize the importance of considering the source of sugars when examining their influence on satiety and FI. Previous research has suggested that sugar-sweetened beverages (SSBs) may bypass regulatory control mechanisms for FI, potentially leading to excess EI and subsequent weight gain, prompting recommendations to limit the consumption of SSBs [40,41,42]. However, experimental trials in young men and children have found evidence of caloric compensation for sugars in solutions, given as preloads, at subsequent meals 30 min to 2 h later [5,6,29,30]. In contrast, FI intake was similar after orange juice, low fat milk, and a soft drink at a test meal 2 h later, but all were higher than the water control [43]. In our study, the orange drink resulted in higher lunch and cumulative FI compared to 100% orange juice and the water control when the ad libitum lunch was provided 60 min after treatment consumption. We observed near complete caloric compensation after 100% orange juice consumption, at 84.0%, compared to overconsumption after the orange drink, at −24.7%, suggesting that the source and composition of the beverage and the time to the next meal are important determinants of short-term FI regulation.

The difference in glycemic response between 100% orange juice and the orange drink was due, in part, to the amount of available carbohydrates in the orange drink. Given that treatments were matched for volume and not available carbohydrates, it is not surprising to observe differences in postprandial glycemic response. However, glycemic response was lower after 100% orange juice compared with the orange drink when corrected for grams of available carbohydrates. The mechanism may be due to the high polyphenols content in 100% orange juice, which have been shown to attenuate glycemic response. Previous animal models, in vitro, and in vivo studies have suggested that the presence of polyphenols can influence carbohydrate digestion and absorption by delaying glucose transport [44,45] and inhibiting digestive enzymes such as alpha-amylase [46], resulting in an attenuated glycemic response [47]. A randomized double-blind crossover trial in adults found lower 24 h glucose concentrations after the consumption of grape juice served with a meal, which is high in polyphenols, compared to a polyphenol-free beverage with reduced flavor served with a meal [48]. Additionally, Kerimi et al. [49] found that hesperidin in orange juice attenuated glucose and fructose transport in rats and modulated post-prandial glucose in healthy adults.

The increase in overweight and obesity globally is thought to be substantially influenced by the heightened intake of sugars, as they are hypothesized to elevate EI by stimulating FI and promoting excess consumption [50,51]. For example, it has been suggested that sweetness may increase appetite due to the stimulation of gut taste receptors [52]. However, others have found no effect of sweet taste on FI [7,53]. In the present study, subjective sweetness scores were higher after the orange drink compared to 100% orange juice and water, and FI was higher after orange drink compared to 100% orange juice. We found a positive association between the subjective sweetness of the orange drink and lunch FI. However, there was no association between sweetness and FI after water or 100% orange juice consumption, suggesting other regulatory mechanisms are responsible for the differences in FI. While it is unlikely that sweetness was the primary determinant of FI, it is possible that it contributed to the differences between treatments found in the present study, highlighting the complex interactions affecting FI.

In adults, epidemiological evidence demonstrated that the consumption of 100% orange juice was associated with increased intake of bioactive flavonoids [54]. Moreover, individuals who included 100% orange juice in their diet exhibited lower consumption of added sugars, adhered to higher-quality diets as measured by the Healthy Eating Index, maintained a lower BMI, and had a reduced likelihood of obesity compared to non-consumers [54,55]. In the present study, total day EI was lower after 100% orange juice by approximately 420 kcal compared with the orange drink. While the present study only evaluated EI over one day, short-term changes in caloric intake, if maintained, may influence body weight and body composition [56,57]. A review of previous work has suggested that the consumption of sugar-containing beverages is associated with weight gain through adding calories to the diet and promoting excessive caloric intake [58]. However, findings from the present study reveal a larger difference in caloric intake when consuming 100% orange juice as a morning preload compared to the orange drink. These findings may help support the evidence from a recent meta-analysis evaluating the effects of different sources of free sugars on adiposity, suggesting that moderate consumption of 100% fruit juice has no adverse effects on body weight [18].

Our findings contribute to a growing body of literature suggesting that consumption of 100% orange juice suppresses FI and may protect against obesity risk in adults [54,55], and further highlights the importance of examining the independent effects of different sugar-containing beverages. Although we examined the effects of consuming ecologically relevant sugar-containing beverages in isolation on satiety, short-term FI, and glycemic response in normal adults, there are several limitations. First, we did not measure satiety hormone responses (e.g., glucagon-like peptide-1, cholecystokinin, and ghrelin), which may have provided clarity on how 100% orange juice and SSBs affect FI regulatory systems. Second, we only included adults with a normal BMI. Future studies should confirm the present study findings in other populations, such as older adults, children and adolescents, and those living with diabetes or obesity, who may respond differently to the test beverages compared with younger, normal-weight adults. Third, we did not consider the effects of biological sex on FI regulation. Sex-based differences in postprandial hormone responses have been reported [59,60] and women have been shown to exhibit more restrained eating behaviors compared with men [61,62], which suggests the need for further examination of sex-based differences. Fourth, given the diversity of commercially available SSBs in the food supply and differences in the ratio of glucose to fructose, we cannot conclude that all SSBs would behave similarly to the SSB selected for this study. Lastly, while short-term changes could influence longitudinal trajectories for these outcomes, our study only measured intraday FI and glycemic response and cannot draw conclusions about whether these findings translate longitudinally. Experimental studies are recommended to examine the longer-term effects of 100% orange juice consumption on components of energy balance and glycemic regulation in adults.

## 5. Conclusions

In conclusion, consumption of 100% orange juice as a preload resulted in higher caloric compensation, lower total daily EI, and lower blood glucose concentrations compared to the orange drink. Future longitudinal studies are needed to assess whether the habitual replacement of SSBs with 100% orange juice may contribute to healthier body weights and improved glycemic control.

## Figures and Tables

**Table 1:** Baseline characteristics of test participants.

Variable	All Participants (*n* = 36 *)
Age (years)	26.8 ± 0.9
Body weight (kg)	65.1 ± 1.8
Height (cm)	171.6 ± 1.8
Body Mass Index (BMI) (kg/m^2^)	22.0 ± 0.3
Fat mass (%) ^1^	23.0 ± 1.3
Fat-free mass (%) ^1^	77.0 ± 1.3

* 18 males and 18 females. ^1^ Estimated using the Bod Pod. All values are presented as means ± SEM.

**Table 2:** Effect of test beverages on lunch food intake (FI), caloric compensation, cumulative FI, rest-of-day energy intake (EI), total EI, water intake, and sensory characteristics.

Variable	Test Beverages (Treatment)			*p*-Value ^†^
	Water	Orange Drink	100% Orange Juice	
Lunch FI (kcal)	1239.9 ± 90.5 ^ab^	1269.3 ± 103.0 ^a^	1151.2 ± 75.5 ^b^	0.054
Caloric Compensation (%)	-	−24.7 ± 40.1	84.0 ± 38.0 *	0.047
Cumulative FI ^1^ (kcal)	1239.9 ± 90.5 ^a^	1388.4 ± 90.4 ^b^	1256.8 ± 90.4 ^a^	0.007
Water intake at lunch (g)	342.4 ± 26.7	340.6 ± 26.4	379.1 ± 26.7	0.17
Rest-of-day EI ^2^ (kcal)	1374.6 ± 132.7	1388.2 ± 98.7	1100.2 ± 90.4	0.04
Total EI ^3^ (kcal)	2974.5 ± 161.2 ^ab^	3136.6 ± 146.8 ^a^	2717.0 ± 104.6 ^b^	0.01
Pleasantness of treatment (mm)	49.2 ± 21.3 ^a^	54.9 ± 4.6 ^a^	72.3 ± 24.1 ^b^	<0.001
Sweetness of treatment (mm)	6.3 ± 2.5 ^a^	79.5 ± 3.2 ^b^	61.4 ± 4.1 ^c^	<0.001

All values represent means ± SEM, *n* = 36. ^†^ *p*-value representing main effect of treatment. Superscripts with different letters within each row denote significant differences (*p* < 0.05; by one-factor ANOVA with Tukey–Kramer’s post hoc test to account for multiple comparisons). * Asterisks represent significant differences between treatments (*p* < 0.05; by paired *t*-test). ^1^ Cumulative FI = test beverage + lunch FI (ad libitum pizza lunch). ^2^ Rest-of-day EI = all FI after ad libitum pizza lunch (assessed using a food record). ^3^ Total EI = standardized breakfast + test beverage + lunch FI + rest-of-day EI.

## Data Availability

The data presented in this study are available upon reasonable request from the corresponding author. The data are not publicly available due to privacy.
